# Biochemical Evaluation by Confirmatory Tests after Unilateral Adrenalectomy for Primary Aldosteronism

**DOI:** 10.1155/2023/5732812

**Published:** 2023-05-24

**Authors:** Shingo Murasawa, Kazunori Kageyama, Mari Usutani, Yuko Asari, Noriko Kinoshita, Yuki Nakada, Yutaka Watanuki, Shinobu Takayasu, Makoto Daimon

**Affiliations:** Department of Endocrinology and Metabolism, Hirosaki University Graduate School of Medicine, Aomori 036-8562, Japan

## Abstract

Primary aldosteronism (PA) is the most common cause of endocrine hypertension. Unilateral PA can be cured using unilateral adrenalectomy (Adx). PA surgery outcome (PASO) criteria, which include clinical and biochemical outcomes, have been proposed to evaluate PA cure after Adx. However, clinical outcomes are often inconsistent with biochemical outcomes. In addition, although confirmatory tests are included as endpoints of biochemical outcomes in the PASO criteria, their clinical usefulness has not yet been established. We evaluated clinical parameters and confirmatory test results before and after Adx in 16 patients with PA and assessed the usefulness of the confirmatory tests. The following were the clinical outcomes after Adx: 37.5% complete success, 62.5% partial success, and 0% absent success. The ratio of biochemical complete success was as follows: 69% aldosterone/renin ratio and basal plasma aldosterone concentration, 19% as assessed by the captopril challenge test, 47% as assessed by the saline infusion test, 30% as assessed by the furosemide upright test, and 100% urine aldosterone. Of these, biochemical complete success was judged in four cases by aldosterone/renin ratio and basal plasma aldosterone concentration, one case by captopril challenge test, five cases by saline infusion test, and one case by furosemide upright test. Although clinical outcomes and urine aldosterone levels improved after Adx, confirmatory tests failed to improve in some cases. The current criteria are not considered useful for biochemical evaluation after Adx. To determine whether additional treatment with mineralocorticoid receptor antagonists is required, more accurate biochemical criteria should be established after Adx.

## 1. Introduction

Primary aldosteronism (PA) is the most common cause of endocrine hypertension. Autonomous hypersecretion of aldosterone results in increased renal tubular reabsorption of sodium (Na) and water and increased potassium (K) excretion, leading to an increased circulating plasma volume. Thus, PA suppresses renin, hypokalemia, and hypertension [1]. The prevalence of PA in hypertensive patients has been reported to be 3.8–12.7% and 1.0–29.8%, in primary care and specialized facilities, respectively [2]. Compared to patients of similar age and blood pressure (BP) with essential hypertension, stroke, cardiac hypertrophy, atrial fibrillation, coronary artery disease, heart failure, and proteinuria are more prevalent in patients with PA [3, 4].

PA is classified into two main types: unilateral (usually adrenal aldosterone-producing adenomas [APA]) and bilateral (idiopathic hyperaldosteronism [IHA]) [1, 5, 6]. Adrenal vein sampling (AVS) is necessary for the diagnosis of unilateral or bilateral aldosterone-producing lesions. In unilateral PA, unilateral adrenalectomy (Adx) is recommended and is expected to normalize excess aldosterone secretion and BP, thereby improving organ damage and preventing progression [7].

In 2017, the primary aldosteronism surgical outcome (PASO) criteria were proposed as a measure to evaluate the outcomes of surgical procedures for PA [8]. In the PASO criteria, after Adx, evaluation is categorized into clinical and biochemical outcomes. Biochemical outcomes have been reported in several studies [8–11] and are evaluated using serum potassium levels, aldosterone/renin ratio (ARR), and confirmatory tests. The criteria were evaluated only in the presence or absence of hypokalemia and/or ARR. However, in PA with normokalemia, the evaluation of biochemical data based only on serum potassium levels after Adx is inadequate. Although confirmatory tests are included in the PASO criteria, the utility of confirmatory tests has not been fully explored after Adx evaluation. The purpose of this study was to investigate the results and utility of confirmatory tests for PA.

## 2. Materials and Methods

We retrospectively studied patients with PA who underwent unilateral adrenalectomy, and the patients were reevaluated using PA confirmatory tests after Adx, at the Hirosaki University Hospital (Hirosaki, Japan) between April 2013 and March 2020. Patients with excess cortisol levels were excluded from the study.

PA was diagnosed according to the guidelines of the Japan Endocrine Society [12]. Plasma renin activity (PRA, ng/mL/h) and plasma aldosterone concentration (PAC, ng/dL) were measured using radioimmunoassay kits (PRA-FR and SPAC-S, respectively; Fujirebio Inc., Tokyo, Japan). Patients who received diuretics, mineralocorticoid receptor antagonists (MRA), beta-adrenergic blockers (*β*B), angiotensin-converting enzyme inhibitors (ACEi), and/or angiotensin receptor blockers (ARB) were evaluated for the PAC/PRA ratio (ARR) after switching from these drugs to other antihypertensive medications, such as calcium channel blockers (CCB) or alpha-adrenergic blockers (*α*B), with regular monitoring of BP (diuretics and MRA were withdrawn over six weeks and *β*B, ACEi, and ARB over two weeks before measurement). The intake of antihypertensive medication was expressed as a defined daily dose (DDD), which assumed the average daily maintenance dose of the antihypertensive medications used for the main indications in adults. The calculation of DDD was based on the World Health Organization's (WHO) Anatomical Therapeutic Chemical/defined daily dose index 2022 (https://www.whocc.no/atc_ddd_index/). The DDD for esaxerenone was not listed; therefore, 2.5 mg of esaxerenone was defined as 1.0 DDD, because this was the initial dosage used in Japan.

PA screening was performed using the criteria ARR > 20 and PAC > 12 ng/dL. After PA screening, all patients underwent at least two of the three confirmatory tests for the diagnosis of PA, including the captopril challenge test (CCT), saline infusion test (SIT), and furosemide upright test (FUT). Each confirmatory test was performed according to the guidelines of the Japan Endocrine Society [12], and tests were started 30 minutes after bed rest. In CCT, PRA and PAC were measured at 0, 60, and 90 min after oral administration of captopril (50 mg). CCT results were judged to be positive when the ARR at 60 or 90 min was greater than 20. In SIT, both PRA and PAC were measured before and 240 min after intravenous infusion of 2 L of 0.9% NaCl. The results of SIT were judged to be positive when the PAC was greater than 6 ng/dL. In FUT, after an intravenous injection of 40 mg furosemide, the patients were maintained in the upright position for 2 h. PRA was measured 2 h after furosemide injection. The results of FUT were judged to be positive when the PRA was greater than 2.0 ng/mL/h. To measure urinary aldosterone (U-Aldo), the patients maintained a 9 g/day salt diet for at least three days, after which 24-hour urine pooling was performed. U-Aldo levels greater than 8 *μ*g/day were judged to be indicative of excessive aldosterone excretion. Potassium supplements were orally administered when the patients showed hypokalemia before the confirmatory tests. Abdominal computed tomography (CT) was also performed to detect adrenal tumors and identify the adrenal veins. An adrenal tumor was defined as a nodule of >5 mm on CT imaging. Autonomous cortisol secretion was evaluated using a 1 mg dexamethasone suppression test according to the diagnostic criteria of the Japan Endocrine Society [13, 14]. Hypokalemia was defined as a potassium level below the reference range.

To determine the localization of aldosterone-producing lesions, adrenal venous sampling (AVS) was performed by experienced radiologists. Tetracosactide acetate (synthetic adrenocorticotropic hormone) was injected as previously reported [6, 12]. Adequate catheterization was considered when adrenal venous cortisol concentration was ≥200 ng/dL. When the lateralized ratio (a higher level of adrenal venous aldosterone/cortisol ratio on one side)/(a lower level of the ratio on the other side) was ≥4.0 or the contralateral ratio (adrenal venous aldosterone/cortisol ratio on the lower level side)/(postcaval aldosterone/cortisol ratio) was <1.0 after synthetic ACTH administration, a unilateral lesion was considered to be present on the side showing a higher level ratio. In a case that did not meet the above criteria, Adx was performed because disease onset was in a patient less than 35 years old, and an adrenal tumor was detected (Patient No. 6 in the Supplemental table 1). AVS was performed, patients were treated with oral MRA, and potassium supplementation was added when hypokalemia persisted even after treatment with MRA.

After AVS, laparoscopic Adx and histological diagnoses were performed at our hospital. All patients had a single tumor, which was defined as an adenoma when larger than 10 mm or as a cortical nodule when smaller than 10 mm. When the hypertension was cured after Adx, antihypertensive medications, including MRA, were discontinued. When hypertension persisted after Adx, CCB or *α*B continued. Patients were evaluated by confirmatory tests and 24-hour urine pooling a few days to eight months after the surgery, as well as preoperatively. Clinical and biochemical outcomes were defined according to the PASO criteria, as shown in Table 1. The categorization of the confirmatory test results according to the PASO criteria is shown in Table 2.

See the Supplementary file for the comprehensive dataset.

## 3. Results

### 3.1. Characteristics of the Study Population before and after Adx

Sixteen patients were enrolled in this study. The patient characteristics before and after Adx are shown in Table 3. The evaluation time after Adx was 4.7 ± 2.2 (0–8) months. The presence of hypokalemia, SBP, DBP, antihypertensive medication, eGFR, PAC, ARR, and U-Aldo levels was significantly lower after Adx, compared to before, and hypokalemia before Adx disappeared. Patient PRA levels were significantly higher after Adx compared to before Adx.

### 3.2. Clinical Outcomes and Biochemical Outcomes

The clinical outcomes following Adx are shown in Table 4. The categories of the clinical complete and the clinical partial were 6 (37.5%) and 10 (62.5%), respectively. The categories of the clinical absence were none. Before Adx, antihypertensive medication showed a significant difference between clinical complete vs. clinical partial (1.1 ± 0.6 vs. 2.1 ± 0.7, *p* < 0.05). After Adx, antihypertensive medication also showed a significant difference between clinical complete vs clinical partial (0.0 ± 0.0 vs. 0.9 ± 0.6, *p* < 0.05). The other parameters did not show any significant differences between the two groups.

### 3.3. Comparison of Patient Characteristics for Each Biochemical Outcome by ARR

Patients were classified according to ARR, and the data were compared between the two groups (Table 5). The categories of ARR complete and partial/absent (partial and absent groups were combined due to the small sample size) were 11 (69%) and 5 (31%), respectively. Accordingly, basal PRA levels after Adx were significantly higher in the ARR complete group than in the ARR partial/absent group (1.1 ± 0.6 vs. 0.4 ± 0.2 ng/mL/h, *p* < 0.05). Basal PAC and ARR levels after Adx were significantly lower in the ARR complete group than in the ARR partial/absent group (10.2 ± 3.6 vs. 15.2 ± 5.1 ng/dL, and 11.4 ± 5.0 vs. 39.8 ± 12.6, respectively, *p* < 0.05).

### 3.4. Results of the Biochemical Outcome by Confirmatory Tests After Adx

The results of the biochemical outcomes, including confirmatory tests after Adx, are shown in Table 6. Clinical results showed six cases of complete success. Of these, biochemical complete success was judged in four cases by ARR and basal PAC, one case by CCT, five cases by SIT, and one case by FUT. Complete success determined by ARR and basal PAC criteria was observed in 11 cases (69%), partial success in one case (6%), and absent success in four cases (25%). Five cases, which were considered partial or absent successes by ARR and basal PAC criteria, were targeted for confirmatory testing using the PASO criteria. Among them, two cases were considered to have complete success with CCT or SIT. On the other hand, 11 cases of complete success by ARR and basal PAC showed one case, five cases, and three cases of complete success by CCT, SIT, and FUT, respectively. The U-Aldo levels showed complete success in all cases after Adx.

## 4. Discussion

We investigated the clinical outcomes of 16 PA patients after Adx. As shown in Table 3, analysis of the overall clinical parameters after Adx showed improvements in many parameters, such as the presence of hypokalemia, SBP, DBP, antihypertensive medication, basal PRA, basal PAC, ARR, and U-Aldo. The reduction in eGFR suggests that aldosterone-induced glomerular hyperfiltration is resolved after Adx [15]. These results indicated that both clinical parameters and excess aldosterone production improved after Adx treatment. The clinical outcomes in our study were similar to those reported in the original report in which the PASO criteria were proposed [8]. As shown in Table 4, in the two groups, based on clinical outcomes, there were no significant differences in clinical parameters other than antihypertensive medication (DDD of antihypertensive medication was included in the classification of clinical outcomes). Both PRA and PAC after Adx tended to be higher in the clinically partial group, although the difference was not statistically significant. However, the lack of statistical significance may be caused by insufficient power due to the small sample size. Otherwise, this result suggests that renin suppression might be weaker in the clinically partial group before Adx and that the effect of excess aldosterone on BP in the clinically partial group was relatively small. This may explain the lack of BP improvement after Adx. Furthermore, biochemical outcomes according to ARR showed no significant differences between the two groups before and after Adx (Table 5). The higher ARR in the ARR partial/absent group reflected a lower basal PRA and higher basal PAC. This result suggests that the difference in ARR before and after Adx failed to correlate with the clinical outcomes.

The rate of complete success for each biochemical outcome, including the confirmatory tests, varied. In the PASO study, confirmatory testing is not recommended when the ARR has been normalized. However, we had been performing loading tests for post-Adx evaluation of patients even with normalized ARRs before the publication of the PASO criteria, and we had observed certain discrepancies in the results. The ARR possesses high sensitivity as a screening test for PA; however, it may yield a false negative result [16]. We simultaneously performed confirmatory testing before loading ARR, namely, basal ARR, in various loading tests. Supplemental data present the test results before loading ARR at the time of CCT (Supplemental table 2). However, supplemental data included those values when basal PRA, PAC, and ARR were measured on a day different from CCT. In other cases, the values of “CCT PRA before loading,” “CCT PAC before loading,” and “CCT ARR before loading” are listed (5 cases: patients 4, 7, 11, 12, and 16). Therefore, 11 out of 16 cases have two basal ARRs listed. Of these 11 cases, the ARR was different among 3 cases (patients 2, 3, and 5). Yozamp et al. reported wide intraindividual variability in the ARR of patients with PA [17], which is not surprising as the ARR showed intraindividual variability in our study as well. Therefore, even after the publication of the PASO criteria, we performed loading tests for post-Adx evaluation even in cases with a normalized ARR. In our study, the confirmatory tests were not consistent even in the five patients judged as a complete success by the ARR and basal PAC criteria (Table 6). Complete success as determined by the ARR and basal PAC criteria was observed in 11 cases (69%). However, the 11 cases of complete success by both ARR and basal PAC showed only one case, five cases, and three cases of complete success by CCT, SIT, and FUT, respectively. In contrast, U-Aldo was assessed as a complete success in all cases, suggesting that Adx improved the daily total secretion of aldosterone. Even in such cases, there are some problems with proving a complete success. First, another residual adrenal gland produced autonomous aldosterone hypersecretion in the case of IHA [18]. Secondly, PA shows abnormal salt responsiveness, which may change after Adx [19]. Third, the criteria for confirmatory tests were not modified after Adx because the patient had only one adrenal gland. It may, therefore, be necessary to reestablish the criteria for confirmatory tests after Adx.

Since PA is diagnosed by biochemical assessment, including confirmatory tests, the evaluation of PA after surgery could also be judged by biochemical assessment. However, it is still unclear how these assessments reflect an adequate state of aldosterone secretion after Adx. For the evaluation of postoperative PA, other criteria, such as the AVIS-2 [9, 10] and CONNsortium criteria [11], have been proposed. In the AVIS-2 criteria, postintervention biochemical failure was defined as the persistence of hypokalemia and an ARR cutoff of 30, with persistently suppressed PRA (<1 ng/mL/h) [9, 10]. However, biochemical outcomes were not defined by the CONNsortium criteria. The PASO criteria first reported confirmatory tests as endpoints for biochemical outcomes. An observational meta-analysis, including 43 studies by Zhou et al., showed that a biochemical cure cannot be ascertained by most of the original studies because postoperative ARR has not been described [20]. Rossi et al. found that CCT significantly improved PAC and ARR after Adx [21]. Dekkers et al. reported that aldosterone levels were suppressed by SIT in 61% of patients after Adx [22]. Miller et al. reported a better improvement of BP in the “cured” group than in the “improved” group when determined by a fludrocortisone suppression test [23]. Moreover, the loading test criteria for the PASO criteria are different from those for PA diagnosis according to the Japan Endocrine Society guidelines. For example, post-Adx ARR of CCT was positive in 3 of the 16 patients according to the diagnostic criteria, and 13 of the 16 patients were in the absent group according to the PASO criteria. We presume that this difference could be attributable to the fact that PRA still tends to be low postoperatively. It is unlikely that only the post-Adx criteria are the same, while the criteria for the loading test in PA diagnosis are not standardized with respect to the guidelines of various societies. In our study, even when the clinical outcomes were judged as a complete success, the assessment of the biochemical outcomes was judged with disparate results. Although the clinical outcomes and urine aldosterone levels improved after Adx, confirmatory tests did not improve. We, therefore, recommend that patients with inconsistent outcomes should be carefully monitored.

Yang et al. discussed the reasons for failure to cure PA after Adx [24] and suggested that either (1) Adx was performed on the wrong side due to limitations of CT scanning or AVS (including the influence of the protocol and the interpretation of results), or (2) only the dominant Adx was done in patients with asymmetrical production of aldosterone. However, the patients in our study were confirmed using both CT and AVS, which showed matching results. Furthermore, in our clinically absent group, strict criteria of LR > 4.0, or CR < 1.0 were adopted, except in only one case (detailed in the Supplemental table 1). Although strict standards were adopted, the second reason postulated by Yang et al. cannot be completely ruled out in this study. When aldosterone hypersecretion exists even after Adx, patients are treated with medications such as a mineralocorticoid receptor antagonists.

Currently, no evidence assessing which loading test criteria should be used postoperatively exists. Considering the differences according to postoperative diagnostic criteria by CCT, at least we can consider the use of postoperative PRA as a criterion inappropriate. The PASO criteria state that “Outcome should be reassessed annually,” owing to residual aldosterone hypersecretion. It is desirable to evaluate whether aldosterone hypersecretion continues as early as possible. Therefore, it would be better to not include PRAs that take time to normalize as criteria. The saline infusion test is based solely on PAC; however, in our study, the complete success rate was only 47%. To resolve this issue, examining optimal parameters by comparing the results of load testing in post-PA surgery patients with long-term prognostic data is warranted in future studies.

This study has some limitations. First, some patients were evaluated for less than three months after Adx. Ishihara et al. reported that an improvement in PAC immediately after Adx may predict a biochemical cure for PA [25]. In the PASO criteria, the first outcome assessment is recommended to be performed more than three months after Adx, and the final outcomes should be assessed 6–12 months later. Wachtel et al. reported that aldosterone levels gradually change over time after Adx for PA [26]; therefore, the definition of “cure” in the PASO criteria should be modified depending on the time course after the operation. Second, the histological diagnoses were classified according to morphological features only. The histopathology of primary aldosteronism (HISTALDO) consensus currently recommends CYP11B12 staining for histological diagnosis of PA [27]. Additionally, the lack of statistical significance may have been caused by insufficient power due to the small sample size.

## 5. Conclusions

The improvement in clinical outcomes after Adx may not mean that aldosterone hypersecretion was completely cured. Although clinical outcomes and urine aldosterone levels improved after Adx, confirmatory tests failed to improve in some cases. If aldosterone hypersecretion exists even after Adx, patients are treated with MRA. To determine whether additional MRA treatment is required, more accurate biochemical criteria must be established after Adx.

## Figures and Tables

**Table 1:** Definition of clinical and biochemical outcomes in PASO criteria.

Clinical outcome	Complete	Normal BP without medication^∗^
	Partial	The same BP before Adx with less medication^∗^ Reduction in BP with either the same or less medication^∗^
	Absent	Unchanged or increased BP with the same or an increased medication^∗^

Biochemical outcome	Complete	Correction of hypokalemia Normalization of ARR In patient with a raised ARR, PAC should be suppressed in confirmatory tests
	Partial	Correction of hypokalemia A raised ARR ≥50% decrease in baseline aldosterone (compared with before Adx) and/or abnormal but improved result after Adx confirmatory tests
	Absent	Persistent hypokalemia A persistent raised ARR Failure to suppress PAC after Adx confirmatory tests

Criteria shown by Ref. [8] are adopted in this study. PASO: primary aldosteronism surgery outcome; BP: blood pressure; medication, antihypertensive medication; ARR: aldosterone-renin ratio; PAC: plasma aldosterone concentration; Adx: adrenalectomy. ^∗^The same and unchanged BP levels are defined as a difference (before Adx vs after Adx) in systolic BP (SBP) of <20 mmHg and diastolic BP (DBP) of <10 mmHg; reduction or increase in BP is defined as a difference in SBP of ≥20 mmHg or DBP of ≥10 mmHg, or both; however, if a change in SBP and an opposing change in DBP are reported, the BP response is defined by the change in SBP. The same medication is defined as a change (decrease or increase) of less than 0.5 times the defined daily dose between before and after Adx; less medication is defined as a decrease of 0.5 or more times the defined daily dose between before and after Adx; and increased medication is defined as an increase of 0.5 or more times the defined daily dose between before and after Adx.

**Table 2:** Definition of the biochemical outcome by confirmatory tests in PASO criteria.

ARR (>20) basal PAC	Complete	ARR < 20
	Partial	ARR ≥ 20 and PAC ≥ 50% decrease
	Absent	*AR*R ≥ 20 and PAC < 50% decrease

CCT	Complete	PAC > 30% decrease
	Partial	ND
	Absent	PAC ≤ 30% decrease

SIT	Complete	PAC < 5.0 ng/dL
	Partial	PAC 5.0-10.0 ng/dL
	Absent	PAC > 10.0 ng/dL

FUT^∗^	Complete	PRA ≥ 2.0 ng/mL/h
	Partial	ND
	Absent	PRA < 2.0 ng/mL/h

U-Aldo^∗∗^	Complete	<10 *μ*g/day
	Partial	10~12 *μ*g/day
	Absent	>2 *μ*g/day

Criteria shown by Ref. [8] are adopted in this study. PASO: primary aldosteronism surgical outcome; ARR: aldosterone-renin ratio; PAC: plasma aldosterone concentration; CCT: captopril challenge test; SIT: saline infusion test; FUT: furosemide upright test; U-Aldo: 24 h urine aldosterone; ND: not defined. ^∗^FUT is not included in the PASO criteria; therefore, the diagnostic criteria of the Japan Endocrine Society are adopted. ^∗∗^U-Aldo is determined according to the criteria for the oral salt loading test in the PASO criteria. BP, estimated glomerular filtration rate (eGFR), basal PRA, and basal PAC levels were measured before and after Adx. Data are presented as the mean ± standard deviation. The statistical significance of the differences in clinical data values was assessed using the Wilcoxon signed-rank test or Fisher's exact test. Statistical significance was set at *p* < 0.05. JMP Pro version 16.2.0 (SAS Institute Japan Ltd., Tokyo, Japan) was used for the statistical analysis.

**Table 3:** Characteristics of patients before and after Adx.

Variables	Before Adx	After Adx	*p* value
Number of patients	16		
Age at Adx (year)	50.7 ± 12.2		
Female/male sex	9/7		
After Adx evaluation time (month)		4.7 ± 2.2	
Presence of hypokalemia (%)	50	0	
SBP (mmHg)	137.7 ± 16.2	120.6 ± 10.1	0.002^∗^
DBP (mmHg)	84.1 ± 11.3	76.4 ± 8.3	0.032^∗^
Medication (defined daily dose)	1.6 ± 0.9	0.6 ± 0.7	<0.001_∗_
eGFR (mL/min/1.73 m^2^)	84.6 ± 16.4	69.3 ± 21.7	<0.001^∗^
Basal PRA (ng/mL/h)	0.3 ± 0.2	0.8 ± 0.6	<0.001^∗^
Basal PAC (ng/dL)	27.1 ± 9.7	11.7 ± 4.4	<0.001^∗^
Basal ARR	138.2 ± 116.0	21.2 ± 15.0	<0.001^∗^
U-Aldo (*μ*g/day)	16.6 ± 6.5	4.9 ± 2.6	<0.001^∗^
AVS LR≧2.6 (yes/no)	15/1		
AVS CR≦1.0 (yes/no)	13/3		
Tumor detected by CT (yes/no)	12/4		
Side for adrenalectomy (left/right)	10/6		
Histological diagnosis (adenoma/cortical nodule)		14/2	

Adx: unilateral adrenalectomy; SBP: systolic blood pressure; DBP: diastolic blood pressure; Medication: antihypertensive medication; eGFR: estimated glomerular filtration rate; PRA: plasma renin activity; PAC: plasma aldosterone concentration; ARR: aldosterone-renin ratio; U-Aldo: 24 h urine aldosterone; AVS: adrenal venous sampling; LR: lateralized ratio; CR: contralateral ratio; CT: computed tomography. Data are presented as means ± standard deviation. The Wilcoxon signed-rank test or Fisher's exact test is performed. ^∗^*p* value < 0.05.

**Table 4:** Clinical outcome.

	Variables	Clinical outcome		*p* value
		Complete	Partial	
	Number of patients	6 (37.5%)	10 (62.5%)	
	Age at Adx (year)	51.2 ± 14.1	50.4 ± 10.9	1.000
	Female/male sex	4/3	5/5	0.633

Before Adx	Presence of hypokalemia (%)	67	40	0.608
	SBP (mmHg)	134.8 ± 9.0	139.4 ± 19.1	0.478
	DBP (mmHg)	82.2 ± 5.2	85.2 ± 13.6	0.356
	Medication (defined daily dose)	1.1 ± 0.6	2.1 ± 0.7	0.022^∗^
	eGFR (mL/min/1.73 m^2^)	88.2 ± 17.6	82.5 ± 15.4	0.626
	Basal PRA (ng/mL/h)	0.3 ± 0.2	0.3 ± 0.1	0.175
	Basal PAC (ng/dL)	30.2 ± 9.3	26.3 ± 9.4	0.148
	Basal ARR	225.4 ± 155.1	87.0 ± 35.4	0.212
	U-Aldo (*μ*g/day)	15.8 ± 7.3	17.2 ± 5.7	0.651
	AVS LR≧2.6 (yes/no)	6/0	9/1	1.000
	AVS CR < 1.0 (yes/no)	5/1	8/2	1.000
	Tumor detected by CT (yes/no)	5/1	7/3	1.000
	Side for Adx (left/right)	3/3	7/3	0.607

After Adx	Evaluation time after Adx (month)	5.3 ± 1.6	4.3 ± 2.5	0.410
	Presence of hypokalemia (%)	0	0	
	SBP (mmHg)	120.2 ± 6.6	120.9 ± 11.7	1.000
	DBP (mmHg)	76.7 ± 7.7	76.3 ± 8.7	0.956
	Medication (defined daily dose)	0.0 ± 0.0	0.9 ± 0.6	0.001^∗^
	eGFR (mL/min/1.73 m2)	71.5 ± 27.3	68.0 ± 17.3	0.704
	Basal PRA (ng/mL/h)	0.6 ± 0.5	1.1 ± 0.6	0.057
	Basal PAC (ng/dL)	8.9 ± 2.7	13.5 ± 4.5	0.058
	Basal ARR	25.8 ± 19.1	17.0 ± 10.8	0.481
	U-Aldo (*μ*g/day)	3.8 ± 2.6	5.6 ± 2.3	0.175
	Histological diagnosis (adenoma/cortical nodule)	5/1	9/1	1.000

Adx: unilateral adrenalectomy; SBP: systolic blood pressure; DBP: diastolic blood pressure; Medication: antihypertensive medication; eGFR: estimated glomerular filtration rate; PRA: plasma renin activity; PAC: plasma aldosterone concentration; ARR: aldosterone-renin ratio; U-Aldo: 24 h urine aldosterone; AVS: adrenal venous sampling; LR: lateralized ratio; CR: contralateral ratio; CT: computed tomography. Data are presented as means ± standard deviation. The Wilcoxon signed-rank test or Fisher's exact test is performed. ^∗^*p* value < 0.05.

**Table 5:** Patient characteristics of the biochemical outcome by ARR.

	Variables	Biochemical outcome by ARR		*p* value
		ARR complete	ARR partial/absent	
	Number of patients	11 (69%)	5 (31%)	
	Age at Adx (year)	52.6 ± 14.0	46.4 ± 8.6	0.427

Before Adx	Female/male sex	6/5	3/2	0.635
	Presence of hypokalemia (%)	63	20	0.141
	SBP (mmHg)	139.9 ± 19.4	132.8 ± 8.0	0.494
	DBP (mmHg)	84.6 ± 13.2	82.8 ± 8.7	0.821
	Medication (defined daily dose)	1.8 ± 1.0	1.7 ± 0.5	0.568
	eGFR (mL/min/1.73 m2)	82.4 ± 18.4	89.5 ± 14.1	0.428
	Basal PRA (ng/mL/h)	0.3 ± 0.2	0.3 ± 0.2	0.642
	Basal PAC (ng/dL)	29.1 ± 9.4	24.9 ± 11.4	0.257
	Basal ARR	135.5 ± 104.5	146.5 ± 172.6	0.821
	U-Aldo (*μ*g/day)	14.8 ± 4.3	19.9 ± 9.5	0.350
	AVS LR≧2.6 (yes/no)	10/1	5/0	0.688
	AVS CR < 1.0 (yes/no)	10/1	3/2	0.156
	Tumor detected by CT (yes/no)	8/3	4/1	1.000
	Side for Adx (left/right)	5/6	5/0	0.058

After Adx	Evaluation time after Adx (month)	5.1 ± 2.2	3.8 ± 2.6	0.422
	Histological diagnosis (adenoma/cortical nodule)	9/2	5/0	0.458
	Presence of hypokalemia (%)	0	0	1.000
	SBP (mmHg)	122.0 ± 11.5	117.6 ± 8.0	0.331
	DBP (mmHg)	77.1 ± 9.7	75.0 ± 6.4	0.909
	Medication (defined daily dose)	0.7 ± 0.8	0.3 ± 0.3	0.950
	eGFR (mL/min/1.73 m^2^)	68.2 ± 24.0	71.7 ± 20.6	0.734
	Basal PRA (ng/mL/h)	1.1 ± 0.6	0.4 ± 0.2	0.020^∗^
	Basal PAC (ng/dL)	10.2 ± 3.6	15.2 ± 5.1	0.047^∗^
	Basal ARR	11.4 ± 5.0	39.8 ± 12.6	0.002^∗^
	U-Aldo (*μ*g/day)	4.5 ± 2.9	5.9 ± 2.1	0.428

Adx: unilateral adrenalectomy; SBP: systolic blood pressure; DBP: diastolic blood pressure; Medication: antihypertensive medication; eGFR: estimated glomerular filtration rate; PRA: plasma renin activity; PAC: plasma aldosterone concentration; ARR: aldosterone-renin ratio; U-Aldo: 24 h urine aldosterone; AVS: adrenal venous sampling; LR: lateralized ratio; CR: contralateral ratio; CT: computed tomography. Data are presented as means ± standard deviation. The Wilcoxon signed-rank test or Fisher's exact test is performed. ^∗^*p* value < 0.05.

**Table 6:** Results of clinical and biochemical outcomes by confirmatory tests after Adx.

Clinical outcome	No.	ARR and basal PAC	CCT	SIT	FUT	U-Aldo
Complete	**1**	**Partial**	**Complete**	**Partial/absent**	**Partial/absent**	**Complete**
	2	Complete	Partial/absent	Complete	Partial/absent	Complete
	3	Complete	Partial/absent	Complete	Partial/absent	Complete
	4	Complete	Partial/absent	Complete	NA	Complete
	**5**	**Absent**	**Partial/absent**	**Complete**	**Partial/absent**	**Complete**
	6	Complete	Partial/absent	Complete	Complete	Complete

Partial	7	Complete	Partial/absent	Complete	Partial/absent	Complete
	8	Complete	Partial/absent	Partial/absent	NA	Complete
	9	Complete	Complete	Partial/absent	NA	Complete
	**10**	**Absent**	**Complete**	**Partial/absent**	**Partial/absent**	**Complete**
	11	Complete	Partial/absent	Partial/absent	Complete	Complete
	**12**	**Absent**	**Partial/absent**	**Complete**	**NA**	**Complete**
	**13**	**Absent**	**Partial/absent**	**Partial/absent**	**NA**	**Complete**
	14	Complete	Partial/absent	Partial/absent	NA	Complete
	15	Complete	Partial/absent	NA	Partial/absent	Complete
	16	Complete	Partial/absent	Partial/absent	Complete	Complete

Ratio of complete		69% (11/16)	19% (3/16)	47% (7/15)	30% (3/10)	100% (16/16)

Adx: unilateral adrenalectomy; ARR: aldosterone-renin ratio; PAC: plasma aldosterone concentration; CCT: captopril challenge test; SIT: saline infusion test; FUT: furosemide upright test; U-Aldo: 24 h urine aldosterone; NA: not available. The cases targeted for confirmatory testing in the PASO (primary aldosteronism surgical outcome) criteria are shown in bold.

## Data Availability

The data used to support the findings of this study are included within the supplementary information file.

## Supplementary Materials

Supplementary Materials Supplemental table 1: characteristics of each patient before Adx. Supplemental table 2: characteristics of each patient after Adx.

## References

[1] Stowasser M., Gordon R. D. (2016). Primary aldosteronism: changing definitions and new concepts of physiology and pathophysiology both inside and outside the kidney. *Physiological Reviews*.

[2] Käyser S. C., Dekkers T., Groenewoud H. J. (2016). Study heterogeneity and estimation of prevalence of primary aldosteronism: a systematic review and meta-regression analysis. *The Journal of Clinical Endocrinology and Metabolism*.

[3] Ohno Y., Sone M., Inagaki N. (2018). Prevalence of cardiovascular disease and its risk factors in primary aldosteronism. *Hypertension*.

[4] Naruse M., Katabami T., Shibata H. (2022). Japan Endocrine Society clinical practice guideline for the diagnosis and management of primary aldosteronism 2021. *Endocrine Journal*.

[5] Umemura S., Arima H., Arima S. (2019). The Japanese Society of Hypertension guidelines for the management of hypertension (JSH 2019). *Hypertension Research*.

[6] Funder J. W., Carey R. M., Franco Mantero M. (2016). The management of primary aldosteronism: case detection, diagnosis, and treatment: an Endocrine Society clinical practice guideline. *The Journal of Clinical Endocrinology and Metabolism*.

[7] Umakoshi H., Tsuiki M., Yokomoto-Umakoshi M. (2018). Correlation between lateralization index of adrenal venous sampling and standardized outcome in primary aldosteronism. *Journal of the Endocrine Society*.

[8] Williams T. A., Lenders J. W. M., Mulatero P. (2017). Outcomes after adrenalectomy for unilateral primary aldosteronism: an international consensus on outcome measures and analysis of remission rates in an international cohort. *The Lancet Diabetes and Endocrinology*.

[9] Rossitto G., Amar L., Azizi M. (2020). Subtyping of primary aldosteronism in the AVIS-2 study: assessment of selectivity and lateralization. *The Journal of Clinical Endocrinology & Metabolism*.

[10] Rossi G. P., Rossitto G., Amar L. (2019). Clinical outcomes of 1625 patients with primary aldosteronism subtyped with adrenal vein sampling. *Hypertension*.

[11] Vorselaars W. M. C. M., Nell S., Postma E. L. (2019). Clinical outcomes after unilateral adrenalectomy for primary aldosteronism. *JAMA Surgery*.

[12] Nishikawa T., Omura M., Satoh F. (2011). Guidelines for the diagnosis and treatment of primary aldosteronism -the Japan Endocrine Society 2009-. *Endocrine Journal*.

[13] Akehi Y., Kawate H., Murase K. (2013). Proposed diagnostic criteria for subclinical Cushing’s syndrome associated with adrenal incidentaloma. *Endocrine Journal*.

[14] Yanase T., Oki Y., Katabami T. (2018). New diagnostic criteria of adrenal subclinical Cushing’s syndrome: opinion from the Japan Endocrine Society. *Endocrine Journal*.

[15] Kramers B. J., Kramers C., Lenders J. W. M., Deinum J. (2017). Effects of treating primary aldosteronism on renal function. *Journal of Clinical Hypertension*.

[16] Rossi E., Regolisti G., Negro A., Sani C., Davoli S., Perazzoli F. (2002). High prevalence of primary aldosteronism using postcaptopril plasma aldosterone to renin ratio as a screening test among Italian hypertensives. *American Journal of Hypertension*.

[17] Yozamp N., Hundemer G. L., Moussa M. (2021). Intra-individual variability of aldosterone concentrations in primary aldosteronism: implications for case detection. *Hypertension*.

[18] Zacharieva S., Orbetzova M., Elenkova A. (2006). Diurnal blood pressure pattern in patients with primary aldosteronism. *Journal of Endocrinological Investigation*.

[19] Catena C., Colussi G. L., Novello M. (2016). Dietary salt intake is a determinant of cardiac changes after treatment of primary aldosteronism. *Hypertension*.

[20] Zhou Y., Zhang M., Ke S., Liu L. (2017). Hypertension outcomes of adrenalectomy in patients with primary aldosteronism: a systematic review and meta-analysis. *BMC Endocrine Disorders*.

[21] Rossi G. P., Pitter G., Bernante P., Motta R., Feltrin G., Miotto D. (2008). Adrenal vein sampling for primary aldosteronism: the assessment of selectivity and lateralization of aldosterone excess baseline and after adrenocorticotropic hormone (ACTH) stimulation. *Journal of Hypertension*.

[22] Dekkers T., Prejbisz A., Schultze Kool L. J. (2016). Adrenal vein sampling versus CT scan to determine treatment in primary aldosteronism: an outcome-based randomised diagnostic trial. *The Lancet Diabetes and Endocrinology*.

[23] Miller B. S., Turcu A. F., Nanba A. T. (2018). Refining the definitions of biochemical and clinical cure for primary aldosteronism using the primary aldosteronism surgical outcome (PASO) classification system. *World Journal of Surgery*.

[24] Yang Y., Reincke M., Williams T. A. (2019). Treatment of unilateral PA by adrenalectomy: potential reasons for incomplete biochemical cure. *Experimental and Clinical Endocrinology & Diabetes*.

[25] Ishihara Y., Umakoshi H., Kaneko H. (2022). Prediction of long-term biochemical cure in patients with unilateral primary hyperaldosteronism treated surgically based on the early post-operative plasma aldosterone value. *Endocrine Journal*.

[26] Wachtel H., Cerullo I., Bartlett E. K. (2014). Long-term blood pressure control in patients undergoing adrenalectomy for primary hyperaldosteronism. *Surgery*.

[27] Williams T. A., Gomez-Sanchez C. E., Rainey W. E. (2021). International histopathology consensus for unilateral primary aldosteronism. *The Journal of Clinical Endocrinology and Metabolism*.

