# Supplementary material for: Biochemical Evaluation by Confirmatory Tests after Unilateral Adrenalectomy for Primary Aldosteronism
Source: J Renin Angiotensin Aldosterone Syst. 2023 May 24;2023:5732812. doi: 10.1155/2023/5732812 (PMC10232090; doi:10.1155/2023/5732812)
Supplement: Supplementary Materials — Supplemental table 1: characteristics of each patient before Adx. Supplemental table 2: characteristics of each patient after Adx. [file 5732812.f1.pdf]

Supplemental table 1. Characteristics of each patients before Adx

Clinical outcome	No.	Age at Adx (year)	Female/Male sex	Presence of hypokalemia (Yes/No)	SBP (mmHg)	DBP (mmHg)	Medication (defined daily dose)	eGFR (mL/min/1.73 m^2)	Basal PRA (ng/mL/h)	Bssal PAC (ng/dL)	Basal ARR	CCT PRA before loading (ng/mL/h)	CCT PAC before loading (ng/dL)	CCT ARR before loading	CCT PAC after loading (ng/dL)	CCT ARR after loading	CCT Ratio of PAC after to before loading (%)	SIT PAC (ng/dL)	FUT PRA (ng/mL/h)	U-Aldo (μg/day)	AVS LR	AVS CR	Tumor detected by CT (Yes/No)
Complete	1	51	Female	Yes	127	75	1.3	91.5	0.1	45.1	451.0	0.2	34.0	170.0	51.6	258.0	151.8	43.6	0.1	28.6	5.8	0.4	Yes
	2	47	Female	Yes	124	78	2.0	111.7	0.1	34.7	347.0	0.1	20.9	209.0	30.5	305.0	145.9	33.1	0.2	18.0	33.0	0.6	Yes
	3	65	Male	Yes	127	85	1.0	69.0	0.2	33.2	166.0	0.1	29.0	290.0	27.2	136.0	93.8	12.2	0.2	20.2	8.5	0.3	Yes
	4	73	Female	Yes	145	84	1.4	62.1	0.1	30.6	306.0	NA	NA	NA	NA	NA	NA	34.0	NA	8.3	20.3	0.2	Yes
	5	32	Female	No	145	91	1.0	103.9	0.3	20.4	60.8	0.3	17.0	56.7	15.7	52.7	92.4	7.8	0.3	11.5	4.8	1.6	Yes
	6	39	Male	No	141	80	0.0	90.9	0.8	17.3	21.6	0.2	10.6	53.0	9.0	47.5	84.9	3.4	1.9	8.1	3.4	0.6	No
Partial	7	62	Male	No	113	69	2.3	64.5	0.3	13.8	46.0	0.5	11.8	23.6	9.6	12.6	81.4	6.9	1.4	NA	1.7	0.9	No
	8	52	Female	Yes	112	60	2.5	64.1	0.3	47.2	157.3	0.2	41.9	209.5	48.7	454.0	116.2	37.5	NA	18.3	18.2	0.3	Yes
	9	59	Female	Yes	146	94	3.1	71.6	0.2	25.1	125.5	0.2	25.1	125.5	31.1	155.5	123.9	26.3	NA	17.3	220.2	0.4	Yes
	10	45	Male	No	128	72	2.0	99.9	0.2	21.3	106.5	0.3	20.7	69.0	14.0	48.7	67.6	7.5	1.4	19.0	3.3	1.3	Yes
	11	46	Male	Yes	145	94	2.5	113.8	0.3	22.7	75.7	0.2	38.6	193.0	50.2	251.0	130.1	17.8	0.5	15.0	28.6	0.2	Yes
	12	51	Female	No	127	87	2.0	68.5	0.7	21.1	30.1	0.6	17.9	29.8	16.6	27.7	92.7	16.3	1.1	9.8	2.7	0.8	Yes
	13	53	Male	No	137	89	2.0	83.5	0.2	16.8	84.0	0.3	33.5	111.7	23.0	69.0	68.7	14.0	NA	30.4	4.5	0.7	No
	14	69	Female	No	175	86	2.7	81.8	0.3	30.5	101.7	0.3	34.3	114.3	39.7	198.5	115.7	25.2	NA	13.0	3.2	0.9	Yes
	15	36	Female	No	150	92	0.7	92.0	0.5	37.2	74.4	0.3	24.0	80.0	17.2	42.0	71.7	NA	0.9	NA	4.9	1.5	No
	16	31	Male	Yes	161	109	1.3	85.1	0.4	27.7	69.3	NA	NA	NA	NA	NA	NA	16.0	NA	15.0	5.6	0.2	Yes

Adx, unilateral adrenalectomy; SBP, systolic blood pressure; DBP, diastolic blood pressure; medication, antihypertensive medication; eGFR, estimated glomerular filtration rate; PRA, plasma renin activity; PAC, plasma aldosterone concentration; ARR, aldosterone-renin ratio; CCT, captopril challenge test; SIT, saline infusion test; FUT, furosemide upright test; U-Aldo, 24 hr urine aldosterone; AVS, adrenal venous sampling; LR, lateralized ratio; CR, contralateral ratio; CT, computed tomography; NA, not available.

Supplemental table 2. Characteristics of each patients after Adx

Clinical outcome	No.	Adx side (Left/Right)	Histological diagnosis (Adenoma/Cortical nodule)	Post-Adx evaluation time (month)	SBP (mmHg)	DBP (mmHg)	Medication (defined daily dose)	eGFR (mL/min/1.73 m^2)	Basal PRA (ng/mL/h)	Basal PAC (ng/dL)	Ratio of PAC (post-Adx to pre-Adx) (%)	Basal ARR	CCT PRA before loading (ng/mL/h)	CCT PAC before loading (ng/dL))	CCT ARR before loading	CCT PAC after loading (ng/dL)	CCT ARR	CCT Ratio of PAC after to before loading (%)	SIT PAC (ng/dL)	FUT PRA (ng/mL/h)	U-Aldo (μg/day)
Complete	1	Left	Adenoma	4	126	81	0.0	56.9	0.2	11.9	26.4	59.5	0.1	14.6	146.0	9.9	53.0	32.2	6.8	0.7	4.3
	2	Right	Adenoma	8	123	76	0.0	94.5	0.4	7.0	20.1	17.5	0.1	10.2	102.0	7.9	41.3	22.5	3.2	0.8	3.8
	3	Left	Adenoma	4	130	90	0.0	53.6	0.3	5.3	16.0	17.7	0.3	6.5	21.7	5.6	18.6	14.2	<1.0	0.5	0.9
	4	Right	Adenoma	4	115	65	0.0	27.3	1.6	7.5	24.6	4.7	1.6	7.5	4.7	6.3	0.5	16.7	3.0	NA	0.0
	5	Left	Adenoma	7	115	72	0.0	98.1	0.3	12.9	63.2	43.0	0.5	7.6	15.2	7.0	7.0	8.4	<1.0	0.1	6.9
	6	Right	Cortical nodule	5	112	76	0.0	98.6	0.7	8.6	49.9	12.3	0.9	7.5	8.3	5.7	2.8	23.7	3.4	3.3	6.8
Partial	7	Left	Adenoma	6	114	78	1.0	47.3	1.5	8.4	61.2	5.6	1.5	8.4	5.6	7.3	2.4	13.2	4.0	1.7	7.5
	8	Right	Adenoma	2	101	65	1.0	55.8	1.0	8.0	16.9	8.0	0.8	8.5	10.6	9.8	8.9	-15.3	5.8	NA	0.6
	9	Left	Adenoma	8	143	97	0.3	50.9	1.1	11.6	46.2	10.5	1.0	10.8	10.8	6.5	4.7	39.7	5.2	NA	5.1
	10	Left	Adenoma	5	117	81	0.7	89.1	0.4	14.3	67.1	35.8	0.2	8.8	44.0	5.9	58.6	33.2	5.7	1.8	7.2
	11	Right	Adenoma	3	122	69	0.5	100.2	0.9	12.8	56.4	14.2	0.9	12.8	14.2	12.2	2.1	4.7	9.6	3.9	6.3
	12	Left	Adenoma	0	124	75	0.5	52.1	0.5	12.8	60.7	25.6	0.5	12.8	25.6	10.9	18.2	14.8	4.4	NA	3.1
	13	Left	Adenoma	3	106	66	0.5	62.1	0.7	24.3	144.6	35.3	0.7	24.3	34.7	20.4	13.1	16.0	7.4	NA	7.9
	14	Left	Adenoma	5	134	76	2.7	81.8	2.5	15.9	52.1	6.4	0.6	9.0	15.0	7.9	17.7	12.7	6.9	NA	6.2
	15	Left	Cortical nodule	3	122	80	0.7	79.4	0.9	16.3	43.8	18.1	0.7	11.7	16.7	10.0	7.2	14.9	NA	1.6	8.0
	16	Right	Adenoma	8	126	76	1.3	61.6	1.0	10.8	39.0	10.8	1.0	10.8	10.8	9.8	4.4	9.1	8.6	5.5	4.3

Adx, unilateral adrenalectomy; SBP, systolic blood pressure; DBP, diastolic blood pressure; medication, antihypertensive medication; eGFR, estimated glomerular filtration rate; PRA, plasma renin activity; PAC, plasma aldosterone concentration; ARR, aldosterone-renin ratio; CCT, captopril challenge test; SIT, saline infusion test; FUT, furosemide upright test; U-Aldo, 24 hr urine aldosterone; NA, not available.
